# Sensitivity and Specificity of a Revised Version of the TRACK-MS Screening Battery for Early Detection of Cognitive Impairment in Patients with Multiple Sclerosis

**DOI:** 10.3390/biomedicines13081902

**Published:** 2025-08-04

**Authors:** Luisa T. Balz, Ingo Uttner, Daniela Taranu, Deborah K. Erhart, Tanja Fangerau, Stefanie Jung, Herbert Schreiber, Makbule Senel, Ioannis Vardakas, Dorothée E. Lulé, Hayrettin Tumani

**Affiliations:** 1 Department of Neurology, Faculty of Medicine, Ulm University, D-89081 Ulm, Germany; luisa.balz@uni-ulm.de (L.T.B.); ingo.uttner@uni-ulm.de (I.U.); daniela.taranu@uni-ulm.de (D.T.); deborah.erhart@uni-ulm.de (D.K.E.); tanja.fangerau@rku.de (T.F.); stefanie.jung@rku.de (S.J.); makbule.senel@uni-ulm.de (M.S.); ioannis.vardakas@uni-ulm.de (I.V.); 2 Neurological Practice Center, Neuropoint Academy & NTD, D-89073 Ulm, Germany; schreiber@neurologie-ulm.de

**Keywords:** multiple sclerosis, cognitive screening, sensitivity, specificity

## Abstract

**Background/Objectives**: Cognitive impairment is one of the most common and debilitating clinical features of Multiple Sclerosis (MS). Neuropsychological assessment, however, is time-consuming and requires personal resources, so, due to limited resources in daily clinical practice, information on cognitive profiles is often lacking, despite its high prognostic relevance. Time-saving and effective tools are required to bridge this gap. This study evaluates the sensitivity and specificity of a revised version of TRACK-MS (TRACK-MS-R), a recently published screening tool to identify cognitive impairment in MS in a fast and reliable way, offering a balance between efficiency and diagnostic yield for the individual patient. **Methods**: In this prospective cross-sectional study, 102 MS patients and 94 age-, sex-, and education-matched healthy controls (HC) completed an extensive neuropsychological assessment, including TRACK-MS-R, to test for cognitive processing speed (Symbol Digit Modalities Test, SDMT) and verbal fluency (Regensburger Word Fluency Test, RWT). Sensitivity of TRACK-MS-R was assessed by using the BICAMS-M battery as a reference, and specificity was determined by comparing MS patients to HC. **Results**: TRACK-MS-R demonstrated high sensitivity (97.44%) when compared to the gold standard as represented by BICAMS-M for early and accurately detecting cognitive impairment in MS patients. Additionally, as a potential cognitive marker, TRACK-MS-R showed a specificity of 82.98% in distinguishing MS patients from healthy controls. **Conclusions**: TRACK-MS-R proves to be a highly sensitive and time-efficient screening tool for detecting cognitive impairment in patients with MS, while demonstrating good specificity compared to HC. Whereas high sensitivity is a prerequisite for a valid screening tool, its relatively modest specificity compared to BICAMS-M (62.9%) calls for caution in interpreting standalone results but instead indicates more extensive neuropsychological testing. Its briefness and diagnostic accuracy support its implementation in routine clinical practice, particularly in time-constrained settings.

## 1. Introduction

Cognitive impairment is considered one of the most common and debilitating sequelae of Multiple Sclerosis (MS), affecting approximately 40–70% of patients throughout the disease course [1,2,3]. It closely interferes with various fields of daily life, including quality of life [4], employment status, and social functioning [5]. Furthermore, tracking cognitive functioning is crucial for monitoring disease progression [6,7,8,9,10,11,12]. Yet, cognitive screening is still underutilized and often not implemented in daily clinical MS care due to time constraints and limited workforce [13]. Current efforts highlight the need to incorporate routine cognitive assessment into MS clinics and practices, including clinical trials [14,15,16]. However, there are gaps in standardized methodological approaches applicable to both clinical and non-clinical settings. Country-specific versions of the Brief International Cognitive Assessment for MS (BICAMS-M) screening battery are the current gold standard for cognitive screening in MS and have been proposed as a practical and validated tool in clinical settings [17,18]. BICAMS-M consists of the Verbal Learning and Memory Test (VLMT; episodic memory) [19], the revised version of the Brief Visuospatial Memory Test (BVMT-R; visual episodic memory) [20], and the Symbol Digit Modalities Test (SDMT; cognitive processing speed) [21] and takes approximately 15–20 min to administer. Yet, even this relatively short test battery may pose challenges in high-throughput clinical settings and out-of-hospital neurologist’s offices in town. Even more, BICAMS-M does not fully capture all cognitive domains relevant to MS, particularly lacking measures of cognitive flexibility as part of executive functions. Executive functions, including planning, problem solving, inhibition, working memory, cognitive flexibility, and strategic thinking [22,23], are a key cognitive domain affected in MS, with evidence of early impairment in the course of the disease [22,24]. Considering the clinical implications, executive functions are often neglected in brief cognitive screening tests, as they usually require more time for administration. One of the briefest and most widely used executive function measurements is the verbal fluency test for one or two minutes. It is considered a sensitive and valid measure of cognitive flexibility and executive control, which is especially true for the 2nd minute of performance [22,25,26,27,28].

To address these issues, a preliminary study by Taranu et al. [29] recently introduced TRACK-MS, a very brief (approximately 5 min to administer) cognitive screening tool designed to detect cognitive impairment in MS patients. TRACK-MS concentrates on the SDMT and the Controlled Oral Word Association Test (COWAT; verbal fluency) [30], two subtests shown to correlate strongly with the Expanded Disability Status Scale (EDSS) in a German MS cohort [29]. TRACK-MS also distinguished MS patients from healthy controls (HC), demonstrating its potential for efficient cognitive monitoring. However, in the first step of TRACK-MS development, the validation was based on a small clinical sample. Moreover, for the application in German-speaking patients, there is a lack of appropriate normative data for the COWAT, as it is not validated for this language. Finally, there was limited evidence with regard to diagnostic validity, despite sensitivity and specificity being key criteria for a valid and reliable diagnostic marker of cognition in MS [29].

We hereby present a revised version of TRACK-MS, which also uses the SDMT but replaces the original verbal fluency measure with a well-validated, language-adapted German verbal fluency task [31,32]. This adapted version, referred to as TRACK-MS Revised (TRACK-MS-R), preserves the original version’s brevity while incorporating information processing and executive function through a 2 min verbal fluency task, for which many different language-specific norms are available. With this approach a combination of a non-culturally dependent screening battery for cognitive processing speed (SDMT) and fluency task with an additional time-related flexibility factor in executive functioning (2 min verbal fluency) is provided for cross-national comparison. It may thus provide an executive function and language-specific compound marker as a valuable supplement to the current gold standard BICAMS-M (with its major focus on memory domain functioning). While replacing the COWAT with the RWT mainly ensures linguistic and normative appropriateness for German speakers, both tests measure the same fundamental aspect of phonemic verbal fluency. Importantly, the extended 2-min version of the RWT improves sensitivity to executive dysfunction by detecting changes in word production over time, thereby preserving the construct validity and diagnostic accuracy of the original TRACK-MS approach.

We evaluated the diagnostic utility of TRACK-MS-R by (1) assessing its sensitivity and specificity as a cognitive screening tool in MS patients, using the established gold standard BICAMS-M as a reference, and (2) by additionally examining its ability to distinguish MS patients from HC, thereby exploring its potential as a cognitive marker.

## 2. Materials and Methods

### 2.1. Design and Participants

One hundred and two MS patients attending the out- and inpatient clinic at the Department of Neurology at Ulm University were consecutively enrolled in this prospective cross-sectional study, with a 24-month data collection period (2023–2025). Also, ninety-four age-, sex-, and education-matched HC were recruited via advertisements in sports facilities and public locations. Inclusion criteria for patients were a diagnosis of clinically and laboratory-confirmed definite MS according to revised McDonald’s criteria [33], and for all subjects, the ability to comprehend and communicate during assessments. Exclusion criteria included motor, speech, or language impairments affecting test validity; severe psychiatric or cognitive disorders; recent corticosteroid use (patients had to be stable for at least 30 days before data collection, with no corticosteroid treatment or relapse interfering during this period); or unstable clinical status. The study was approved by the ethics committee of the University of Ulm (No. 335/23). All participants gave informed consent prior to their inclusion in the study.

### 2.2. Procedure

All MS patients underwent a comprehensive clinical neurological examination by a trained physician in the clinical setting. Subsequently, participants completed a detailed neuropsychological assessment focusing on memory, attention, executive, and visuospatial functions. In addition, participants reported outcome measures on depression, anxiety, and fatigue, while demographics and clinical data were collected via semi-structured interviews. Physical impairment was measured using the Expanded Disability Status Scale (EDSS with values from 0 = normal neurological function to 10 = death) [34].

### 2.3. Detailed Neuropsychological Assessment

To exclude severe cognitive impairment and to provide a benchmark scale for cognitive function, an extensive neuropsychological assessment was conducted, targeting a broad range of cognitive domains. Verbal short-term and working memory were evaluated using the Digit Span Test from the Wechsler Memory Scale-Revised (WMS-R) [35], while nonverbal short-term and working memory were assessed with the Block-Tapping Test (WMS-R). Verbal and visual episodic memory were addressed using the Verbal Learning and Memory Test (VLMT) [19] and the revised version of the Brief Visuospatial Memory Test (BVMT-R) [20]. Attention domains, including divided attention and incompatibility, were evaluated with the German “Testbatterie zur Aufmerksamkeitsprüfung” (TAP) [36]. Logical reasoning and concept formation were assessed using the Matrices subtest of the German version of the Wechsler Adult Intelligence Scale (WAIS) [37].

### 2.4. TRACK-MS and TRACK-MS-R

The Symbol Digit Modalities Test (SDMT) [21] was employed to measure information processing speed as well as divided and selective attention. Verbal fluency was assessed using both the Controlled Oral Word Association Test (COWAT) [30] and the Regensburger Wortflüssigkeitstest (RWT, phonematic fluency letter “S”), the most widely used German adaptation of verbal fluency measures [31,32].

The ultrashort screening tool TRACK-MS (SDMT + COWAT) was established as a very brief repeatable cognitive tracking tool with comparable test quality criteria as BICAMS-M [29], combining the two tests with the highest validity for MS-specific cognitive changes—the Controlled Oral Word Association Test (COWAT) and SDMT—with an administration time of 5 min.

The revised version of TRACK-MS (TRACK-MS-R, SDMT + RWT, letter “S”) replaces the COWAT with the RWT, which is the German equivalent, and allows a 2-min word generation period using the letter “S”. In contrast, the COWAT requires participants to generate words using the letters “F-A-S” for one minute each. TRACK-MS-R is thus slightly shorter than the original version (about 4 min). Most importantly, normative data for the German-speaking population are available.

### 2.5. MS-Specific Cognitive Screening Batteries

The current gold standard for cognitive screening in MS, the Brief International Cognitive Assessment for MS (BICAMS-M), which was established by the international expert consensus committee to promote and facilitate an international standard, was used in its validated German version [38]. It includes tests of cognitive processing speed (SDMT) and verbal and non-verbal memory (VLMT learning and BVMT-R learning) [17] and takes approximately 20 min to administer.

### 2.6. Depression and Anxiety

Depression and anxiety were documented as patient-reported measures using the Hospital Anxiety and Depression Scale (HADS; 7 items, range 0 to 21 for depression and anxiety, respectively), which is specifically aimed at people with physical illnesses or physical complaints, with cut-off scores for mild (≥8), moderate (≥11), or severe (≥15) [39] depression and anxiety, respectively.

### 2.7. Fatigue

Fatigue was measured using the Fatigue Scale for Motor and Cognitive Functions (FSMC), a 20-item questionnaire evaluating motor and cognitive fatigue. Each item is rated on a 5-point Likert scale (1–5), with cut-offs for motor fatigue: mild (≥22), moderate (≥27), severe (≥32); for cognitive fatigue: mild (≥22), moderate (≥28), severe (≥34) [40].

### 2.8. Statistical Analysis

Statistical analyses were performed using SPSS version 29 (IBM Corp., Armonk, NY, USA). A priori power calculations ensured 80% power to detect medium to large effect sizes (partial η^2^ = 0.06–0.14) with α = 0.05, requiring N = 94 MS patients and N = 94 HC. Gender matching was assessed using the chi-square test. Group differences in cognition, depression, anxiety, and fatigue were analyzed via ANOVA/ANCOVA. A normal distribution can be assumed due to the sample size of N > 30 [41]. Composite scores for BICAMS-M and TRACK-MS-R were calculated by averaging z-standardized subtest scores. Z-standardization was performed using data from 94 HC. Sensitivity and specificity were calculated using a standard 2 × 2 contingency table (TN = true negative, TP = true positive, FP = false positive, FN = false negative), with the following formulas: specificity = TN/(TN + FP) and sensitivity = TP/(TP + FN).

## 3. Results

### 3.1. Demographics and Clinical Data

A total of 196 participants were included, comprising 102 patients with MS and 94 HC. The groups were comparable in terms of age and education. A significantly higher proportion of females was observed in the HC group compared to the MS patients. The majority of MS patients were diagnosed with relapsing-remitting MS (RRMS), and overall patients showed only minor physical impairment, as indicated by a mean EDSS score ≤ 3.5 [42]. Participant characteristics are summarized in Table 1.

### 3.2. Neuropsychological Assessment

After controlling for gender, significant differences between MS patients and HC were observed across most cognitive domains assessed in the extensive neuropsychological test battery (see Table 2). MS patients showed significantly lower performance compared to HC in verbal and nonverbal short-term memory, verbal working memory, verbal and visual episodic memory, attentional functions (including visual divided attention, incompatibility, and cognitive processing speed), and executive functions. No significant group differences were found for nonverbal working memory (Block-Tapping Test backwards), the recognition task of visual episodic memory (BVMT-R recognition), and auditory divided attention (all *F* < 2.56 and *p* > 0.05). These specific subtests are not included in core cognitive test batteries such as TRACK-MS-R or BICAMS-M and are therefore not considered further in the subsequent sensitivity and specificity analyses.

### 3.3. Affective State and Fatigue

MS patients reported significantly greater symptoms of depression and anxiety and higher levels of cognitive and motor fatigue (see Table 2).

### 3.4. Sensitivity and Specificity of TRACK-MS-R vs. Gold Standard BICAMS-M

**Figure 1:**
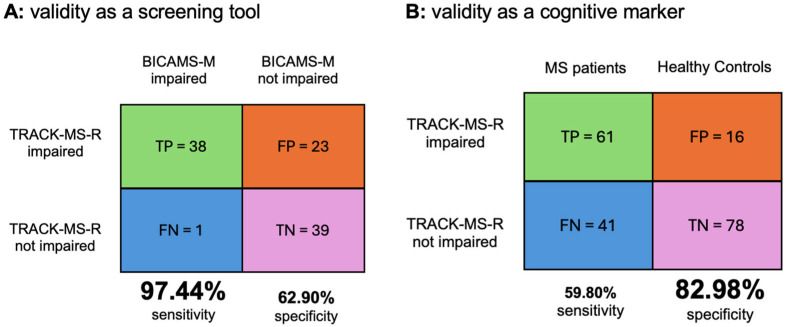
Validity of TRACK-MS-R. Standard 2 × 2 contingency tables illustrating the validity of TRACK-MS-R as a screening tool (**A**) and as a cognitive marker (**B**). The matrices display true positives (TP), false positives (FP), true negatives (TN), and false negatives (FN) to determine sensitivity and specificity. (**A**): classification of MS patients by TRACK-MS-R compared to the gold standard BICAMS-M. (**B**): classification performance of TRACK-MS-R in differentiating MS patients from Heathy Controls. Colors are used solely for visual clarity: green = TP, orange = FP, blue = FN, purple = TN.

To evaluate the utility as a screening tool for cognitive impairment within the MS population, the sensitivity and specificity of TRACK-MS-R were determined using BICAMS-M as the gold standard. The classification was based on the standard 2 × 2 contingency table (see Figure 1). Based on these data, TRACK-MS-R demonstrated a sensitivity of 97.44% and a specificity of 62.90% when compared to the gold standard BICAMS-M (see Figure 1).

### 3.5. Sensitivity and Specificity of TRACK-MS-R as a Cognitive Marker of MS

To further assess the utility of TRACK-MS-R as a potential cognitive marker to classify MS patients as impaired in comparison to HC, a 2 × 2 contingency table was again applied to calculate sensitivity and specificity relative to HC classification (see Figure 1). Based on this classification, TRACK-MS-R yielded a specificity of 82.98% in distinguishing MS patients from HC (see Figure 1) and a sensitivity of 59.80%.

## 4. Discussion

This study evaluated the diagnostic utility of the revised version of the very short test battery TRACK-MS (TRACK-MS-R) for detecting cognitive impairment in patients with MS in routine clinical and doctor practice. We hereby present diagnostic performance metrics such as sensitivity and specificity for the German-speaking population. Results indicate that TRACK-MS-R is a highly sensitive screening tool (97.44%) for identifying cognitive impairment in MS patients, which is often a result of cortical involvement/lesions and more prevalent in progressive disease states [2,43]. TRACK-MS-R demonstrates good specificity (82.98%) when distinguishing MS patients from healthy controls, which allows a more precise definition of MS-specific cognitive changes. The short administration time of a maximum of 5 min further underscores its potential for implementation in clinical routine in hospitals and medical practices, i.e., time-constrained settings [44] compared to the current gold standard BICAMS-M. By confirming and broadening the applicability of TRACK-MS-R in a larger and more representative German-speaking cohort of MS patients and HC, these findings build upon and extend the previous work by Taranu et al. [29], who introduced the original TRACK-MS as a brief and clinically feasible alternative to standard cognitive assessments such as the current gold standard BICAMS-M [17]. In contrast to the initial validation, the current study incorporates a language-adapted verbal fluency task [31], for which extensive normative data for many different languages, including the German-speaking population, are available. The hereby presented diagnostic validation of TRACK-MS-R represents a critical step toward clinical utility, as it allows for reliable and time-efficient identification of cognitively impaired individuals in everyday practice, without sacrificing diagnostic accuracy. As a compound marker of cognitive processing speed and executive functioning, it substantially adds to the current gold standard BICAMS-M. The inclusion of information processing speed and executive functioning as key domains in TRACK-MS-R emphasizes its clinical importance and accounts for its ability to detect early cognitive changes in MS. These functions are closely related to the integrity of frontal and subcortical white matter pathways, which are often affected by inflammatory lesions and neurodegenerative changes typical of MS [45,46,47,48,49]. Neuroimaging studies consistently show that cognitive impairment in MS is linked to both cortical lesions and the disruption of fronto-subcortical circuits involved in executive control and mental flexibility [50,51,52]. Processing speed in particular is one of the most sensitive cognitive domains affected in MS and correlates strongly with lesion burden, white matter disconnection, and regional atrophy in these networks [50,51,52]. These pathophysiological insights strengthen the construct validity of TRACK-MS-R and support its use as a practically feasible screening tool that targets neural systems affected early in MS-related cognitive decline.

As emphasized in recent literature [2,6,53,54,55,56,57,58,59,60,61], our findings align with the urgent need to implement more time-efficient cognitive screening instruments into routine clinical care for MS patients. Due to its high sensitivity, TRACK-MS-R ensures that cognitively impaired MS patients are correctly identified. In clinical practice, this makes TRACK-MS-R particularly useful for “ruling in” individuals who may benefit from more comprehensive neuropsychological evaluation or early cognitive interventions [62,63]. Its greatest utility may lie in early-stage screening, where subtle cognitive changes can guide timely diagnostic clarification or therapeutic adjustments [5,55,58,64]. Moreover, this high sensitivity significantly reduces the risk of underdiagnosis, an ongoing challenge in MS care, where cognitive symptoms often remain unrecognized and thus untreated [3,54,65,66].

While the high sensitivity of TRACK-MS-R supports its utility as an early detection tool, its moderate specificity (62.9% compared to BICAMS-M) indicates that some MS patients without relevant cognitive impairment may screen positive. In routine clinical care, this trade-off may be acceptable when the main goal is early detection of cognitive impairment. However, clinicians should be aware that a positive TRACK-MS-R result may require additional, more elaborate neuropsychological assessment to confirm impairment and determine appropriate interventions.

The good specificity of TRACK-MS-R compared to HC indicates that MS patients are accurately classified as cognitively impaired, minimizing false-negative results. This strengthens the tool’s reliability in distinguishing MS-related cognitive impairment from normal cognitive functioning. As such, TRACK-MS-R holds promise not only for individual-level screening but also as a supportive tool in research contexts. These findings are in line with those reported by Taranu et al. [29], who also demonstrated significant group-level differences using the original TRACK-MS battery in a German MS sample, thereby further validating the clinical and research utility of the TRACK-MS approach.

From a methodological standpoint, replacing the Controlled Oral Word Association Test (COWAT) [30] with the RWT strengthened the construct validity of the verbal fluency domain while enhancing its linguistic and cultural appropriateness for German-speaking populations in specific. Many different language-specific adaptations of the verbal fluency measure are available and may be implemented as required, substantially increasing the wide application of Track-MS-R and making it especially important for international studies. Building on this, the RWT improves the diagnostic validity of TRACK-MS-R by incorporating a full two-minute word generation period. This captures the decline in verbal output associated with controlled retrieval and executive function [67]. In the future, other subtests of the RWT, in addition to assessing formal-lexical fluency, may be added, as the RWT also offers subtests to assess semantic fluency and both formal-lexical and semantic category switching. This allows for a more comprehensive evaluation of executive domains without substantially extending the test administration time. While TRACK-MS-R was validated in a German-speaking population using an adapted and linguistically validated verbal fluency task, future research is needed to evaluate its applicability in broader linguistic and cultural contexts. Given that language-specific versions of the verbal fluency task do exist, TRACK-MS-R could be adapted in a modular way to support cross-national comparisons while maintaining strong psychometric properties. This approach would enable testing and potentially implementing TRACK-MS-R in various international healthcare settings.

The implications of these findings are two-fold. First, due to its brevity and ease of administration, TRACK-MS-R is suitable not only for neuropsychologists or neurologists but also for other healthcare professionals involved in MS care, such as general practitioners or nurses. This broadens its applicability in interdisciplinary settings. Second, the reliable group-level differentiation between MS patients and healthy controls supports its potential use as a cognitive marker in clinical research.

However, certain limitations must be acknowledged: (a) While the sample size was sufficient for cross-sectional diagnostic analyses, future studies need to examine the longitudinal sensitivity of TRACK-MS-R to detect subtle cognitive decline over time, which would provide further evidence for its utility in disease monitoring; (b) Unlike more comprehensive cognitive screening batteries like BICAMS-M, TRACK-MS-R does not directly assess memory functions, so it is not an integral cognitive screening tool. However, the RWT verbal fluency task indirectly captures memory-relevant processes: retrieving words from long-term memory is essential to completing the task. Also, working memory is addressed since patients have to monitor their verbal output to avoid repetition. Therefore, although there are no explicit memory subtests, certain memory-related mechanisms (e.g., working and episodic memory) are still involved [26]; (c) Participants were recruited from a single university hospital and by public advertisements. This convenience sampling may limit the generalizability of findings to broader MS populations, particularly those with lower education levels or more severe physical disability; (d) Although TRACK-MS-R combines a well-known verbal fluency measure (RWT) with existing normative data for the German-speaking population, there are currently no separate normative data for the complete TRACK-MS-R screening tool. This approach was chosen to enable early clinical application and feasibility testing. However, we are currently conducting longitudinal follow-up studies within a German MS cohort to evaluate TRACK-MS and TRACK-MS-R over time. These studies aim to evaluate the sensitivity of TRACK-MS-R in detecting subtle cognitive changes at different disease stages and treatment intervals. As part of this, we are validating both tools across inpatient and outpatient settings and are currently developing specific normative data for TRACK-MS-R. This will enable the creation of reliable change indices, which are crucial for interpreting within-subject changes across different time points. Developing these normative data will further strengthen its diagnostic validity and clinical applicability for future studies.

## 5. Conclusions

In conclusion, TRACK-MS-R emerges as a psychometrically robust, time-efficient, and clinically feasible cognitive screening instrument for MS patients. By demonstrating excellent sensitivity and solid specificity, TRACK-MS-R provides a valid screening alternative to more time-consuming test batteries such as BICAMS-M, without compromising diagnostic accuracy. It may as well be used in addition to BICAMS-M to extend the BICAMS-M memory domain related findings by the executive function domain of Track-MS-R. Its brief administration time, cultural adaptability through validated language-specific norms, and focus on core cognitive domains relevant to MS—namely, processing speed and verbal fluency—render it highly suitable for routine implementation in a broad spectrum of clinical settings, including outpatient care and non-specialist environments. Furthermore, its potential application as a cognitive marker may support its integration into longitudinal monitoring protocols and research frameworks. Future longitudinal studies are warranted to confirm its utility in tracking cognitive trajectories and informing therapeutic decisions over the course of the disease. Overall, TRACK-MS-R represents a significant contribution to the practical assessment of cognitive function in MS, also addressing an unmet need in time-constrained clinical practice.

## Figures and Tables

**Table 1:** Demographics and clinical data.

Characteristics	MS Patients (N = 102)		HC * (N = 94)		Statistics ^a^
	Mean (SD)	N (%)	Mean (SD)	N (%)	
Age (years)	45.49 (13.29)		47.13 (14.11)		*F*(1, 194) = 0.70, *p* = 0.404
Female/Male		61/41 (59.8/40.2)		69/25 (73.4/26.6)	*χ^2^*(1) = 4.05, *p* = 0.044
Years of education	14.61 (2.92)		14.91 (2.85)		*F*(1, 194) = 0.56, *p* = 0.457
EDSS *	2.79 (1.82)				
Time since diagnosis (years)	9.97 (8.09)				
Phenotype					
RRMS *		73 (71.6)			
PPMS *		11 (10.8)			
SPMS *		17 (16.7)			

* EDSS = Expanded Disability Status Scale; RRMS = Relapsing Remitting Multiple Sclerosis; SPMS = Secondary Progressive Multiple Sclerosis; PPMS = Primary Progressive Multiple Sclerosis; HC = Healthy Controls. ^a^ ANOVA or Chi-square test for group comparison; threshold for significant difference with *p* < 0.05.

**Table 2:** Neuropsychological profile, affective state, and fatigue.

Cognitive Domains/PROMs *		MS Patients		HC *		Statistics ^a^		
**Tests**	**N (Cohort)**	**Mean (SD)**	**N (%)**	**Mean (SD)**	**N (%)**	***F***	***p*** **-Value**	**Partial η^2^**
**Verbal short-term memory**								
digit span forward	196	7.00 (1.85)		8.11 (1.65)		9.77	**<0.001**	0.092
**Nonverbal short-term memory**								
block-tapping-test forward	196	8.17 (1.83)		9.39 (1.77)		11.82	**<0.001**	0.109
**Verbal working memory**								
digit span backwards	196	5.75 (1.97)		6.81 (1.86)		8.15	**<0.001**	0.78
**Nonverbal working memory**								
block-tapping-test backwards	196	8.45 (6.70)		10.59 (10.89)		1.40	0.248	0.014
**Verbal episodic memory**								
VLMT * total	196	50.55 (11.45)		59.31 (8.05)		30.16	**<0.001**	0.238
VLMT * delayed recall	196	10.14 (3.94)		12.78 (2.64)		21.10	**<0.001**	0.179
VLMT * recognition	196	13.37 (2.60)		14.48 (0.86)		7.89	**<0.001**	0.076
**Visual episodic memory**								
BVMT-R * total	196	24.55 (8.04)		28.20 (5.49)		6.94	**0.001**	0.067
BVMT-R * delayed recall	196	9.73 (2.88)		10.85 (1.73)		5.96	**0.003**	0.058
BVMT-R * recognition	196	5.65 (0.94)		5.87 (0.42)		2.56	0.080	0.026
**Attentional functions**								
TAP * divided attention (auditory)	190	657.82 (159.65)		613.09 (111.74)		2.47	0.087	0.026
TAP * divided attention (visual)	190	871.54 (214.88)		757.03 (95.60)		11.41	**<0.001**	0.109
TAP * incompatibility	190	573.99 (180.83)		501.68 (109.98)		5.47	**0.005**	0.055
SDMT *	196	50.13 (13.31)		59.77 (9.33)		17.05	**<0.001**	0.150
**Executive functions**								
phonemic verbal fluency (RWT * S)	196	18.99 (7.16)		24.47 (7.01)		14.95	**<0.001**	0.135
phonemic verbal fluency (COWAT *)	196	34.25 (9.95)		43.77 (12.11)		19.22	**<0.001**	0.166
matrices (WAIS *)	193	17.16 (4.81)		19.51 (3.13)		8.38	**<0.001**	0.081
Cognitive fatigue (FSMC *)	196	30.12 (12.05)		18.46 (7.50)		32.46	**<0.001**	0.253
mild			13 (12.7)		18 (19.1)			
moderate			17 (16.7)		9 (9.6)			
severe			44 (43.1)		3 (3.2)			
Motor fatigue (FSMC *)	196	31.83 (11.60)		18.34 (7.31)		46.19	**<0.001**	0.325
mild			10 (9.8)		16 (17)			
moderate			11 (10.8)		10 (10.6)			
severe			57 (55.9)		4 (4.3)			
Depression (HADS-D *)	196	5.81 (4.20)		2.96 (2.64)		16.26	**<0.001**	0.144
mild			20 (19.6)		7 (7.4)			
moderate			14 (13.7)		0 (0)			
severe			2 (2)		0 (0)			
Anxiety (HADS-A *)	196	7.19 (4.48)		4.29 (2.55)		15.06	**<0.001**	0.135
mild			19 (18.6)		7 (7.4)			
moderate			15 (14.7)		0 (0)			
severe			9 (8.8)		0 (0)			

* PROMs = Patient-Reported Outcome Measures; HC = Healthy Controls; VLMT = Verbal Learning Memory Test; BVMT-R = Brief Visuospatial Memory Test—Revised; TAP = Testbatterie zur Aufmerksamkeitsprüfung; SDMT = Symbol Digit Modalities Test; RWT = Regensburger Wortflüssigkeitstest (German Version of Verbal Fluency); COWAT = Controlled Oral Word Association Test; WAIS = Wechsler Adult Intelligence Scale; FSMC = Fatigue Scale for Motor and Cognitive Functions; HADS = Hospital Anxiety and Depression Scale. ^a^ ANCOVA for group comparison, adjusted for gender; threshold for significant difference with *p* < 0.05 (significant results are highlighted in bold).

## Data Availability

The raw data supporting the conclusions of this article will be made available by the authors on request.

## Abbreviations

The following abbreviations are used in this manuscript:

MS	Multiple Sclerosis
RRMS	Relapsing-Remitting Multiple Sclerosis
PPMS	Primary Progressive Multiple Sclerosis
SPMS	Secondary Progressive Multiple Sclerosis
EDSS	Expanded Disability Status Scale
HC	Healthy Controls
BICAMS	Brief International Cognitive Assessment for MS
VLMT	Verbal Learning and Memory Test
BVMT-R	Brief Visuospatial Memory Test—Revised Version
SDMT	Symbol Digit Modalities Test
COWAT	Controlled Oral Word Association Test
WAIS	Wechsler Adult Intelligence Scale
RWT	Regensburger Wortflüssigkeitstest
TAP	Testbatterie zur Aufmerksamkeitsprüfung
WMS-R	Wechsler Memory Scale-Revised
HADS	Hospital Anxiety and Depression Scale
FSMC	Fatigue Scale for Motor and Cognitive Functions
TN	True negative
TP	True positive
FP	False positive
FN	False negative

## References

[1] Chiaravalloti N.D., De Luca J. (2008). Cognitive impairment in multiple sclerosis. Lancet Neurol..

[2] Benedict R.H.B., Amato M.P., DeLuca J., Geurts J.J.G. (2020). Cognitive impairment in multiple sclerosis: Clinical management, MRI, and therapeutic avenues. Lancet Neurol..

[3] Amato M.P., Zipoli V., Portaccio E. (2006). Multiple sclerosis-related cognitive changes: A review of cross-sectional and longitudinal studies. J. Neurol. Sci..

[4] Eijlers A.J.C., van Geest Q., Dekker I., Steenwijk M.D., Meijer K.A., Hulst H.E., Barkhof F., Uitdehaag B.M.J., Schoonheim M.M., Geurts J.J.G. (2018). Predicting cognitive decline in multiple sclerosis: A 5-year follow-up study. Brain.

[5] Macias Islas M.A., Ciampi E. (2019). Assessment and Impact of Cognitive Impairment in Multiple Sclerosis: An Overview. Biomedicines.

[6] Kalb R., Beier M., Benedict R.H., Charvet L., Costello K., Feinstein A., Gingold J., Goverover Y., Halper J., Harris C. (2018). Recommendations for cognitive screening and management in multiple sclerosis care. Mult. Scler..

[7] Benito-Leon J., Morales J.M., Rivera-Navarro J., Mitchell A. (2003). A review about the impact of multiple sclerosis on health-related quality of life. Disabil. Rehabil..

[8] Morrow S.A., Rosehart H., Pantazopoulos K. (2016). Anxiety and Depressive Symptoms Are Associated with Worse Performance on Objective Cognitive Tests in MS. J. Neuropsychiatry Clin. Neurosci..

[9] Ruet A., Deloire M., Hamel D., Ouallet J.C., Petry K., Brochet B. (2013). Cognitive impairment, health-related quality of life and vocational status at early stages of multiple sclerosis: A 7-year longitudinal study. J. Neurol..

[10] De Meo E., Portaccio E., Giorgio A., Ruano L., Goretti B., Niccolai C., Patti F., Chisari C.G., Gallo P., Grossi P. (2021). Identifying the Distinct Cognitive Phenotypes in Multiple Sclerosis. JAMA Neurol..

[11] Wills O., Probst Y. (2024). Towards new perspectives: A scoping review and meta-synthesis to redefine brain health for multiple sclerosis. Eur. J. Neurol..

[12] Delgado-Alvarez A., Delgado-Alonso C., Goudsmit M., Gil M.J., Diez-Cirarda M., Valles-Salgado M., Montero-Escribano P., Hernandez-Lorenzo L., Matias-Guiu J., Matias-Guiu J.A. (2022). Validation of a brief cross-cultural cognitive screening test in Multiple Sclerosis. Mult. Scler. Relat. Disord..

[13] Lechner-Scott J., Agland S., Allan M., Darby D., Diamond K., Merlo D., van der Walt A. (2023). Managing cognitive impairment and its impact in multiple sclerosis: An Australian multidisciplinary perspective. Mult. Scler. Relat. Disord..

[14] Sumowski J.F., Benedict R., Enzinger C., Filippi M., Geurts J.J., Hamalainen P., Hulst H., Inglese M., Leavitt V.M., Rocca M.A. (2018). Cognition in multiple sclerosis: State of the field and priorities for the future. Neurology.

[15] van Dongen L., Westerik B., van der Hiele K., Visser L.H., Schoonheim M.M., Douw L., Twisk J.W.R., de Jong B.A., Geurts J.J.G., Hulst H.E. (2020). Introducing Multiple Screener: An unsupervised digital screening tool for cognitive deficits in MS. Mult. Scler. Relat. Disord..

[16] Wojcik C.M., Beier M., Costello K., DeLuca J., Feinstein A., Goverover Y., Gudesblatt M., Jaworski M., Kalb R., Kostich L. (2019). Computerized neuropsychological assessment devices in multiple sclerosis: A systematic review. Mult. Scler..

[17] Langdon D.W., Amato M.P., Boringa J., Brochet B., Foley F., Fredrikson S., Hamalainen P., Hartung H.P., Krupp L., Penner I.K. (2012). Recommendations for a Brief International Cognitive Assessment for Multiple Sclerosis (BICAMS). Mult. Scler..

[18] Potticary H., Langdon D. (2023). A Systematic Review and Meta-Analysis of the Brief Cognitive Assessment for Multiple Sclerosis (BICAMS) International Validations. J. Clin. Med..

[19] Helmstaedter C., Lendt M., Lux S. (2001). Verbaler Lern-Und Merk-Fähigkeitstest: VLMT: Manual.

[20] Benedict R.H.B., Schretlen D.S., Groninger L., Dobraski M. (1996). Revision of the brief Visuospatial Memory Test: Studies of normal performance, reliability and validity. Psychol. Assess..

[21] Benedict R.H.B., DeLuca J., Phillips G., LaRocca N., Hudson L.D., Rudick R., Consortium M.S.O.A. (2017). Validity of the Symbol Digit Modalities Test as a cognition performance outcome measure for multiple sclerosis. Mult. Scler..

[22] Henry J.D., Beatty W.W. (2006). Verbal fluency deficits in multiple sclerosis. Neuropsychologia.

[23] Godefroy O., Azouvi P., Robert P., Roussel M., LeGall D., Meulemans T., Groupe de Réflexion sur l’Evaluation des Fonctions Exécutives Study Group (2010). Dysexecutive syndrome: Diagnostic criteria and validation study. Ann. Neurol..

[24] Cerezo Garcia M., Martin Plasencia P., Aladro Benito Y. (2015). Alteration profile of executive functions in multiple sclerosis. Acta Neurol. Scand..

[25] Meca-Lallana V., Gascon-Gimenez F., Ginestal-Lopez R.C., Higueras Y., Tellez-Lara N., Carreres-Polo J., Eichau-Madueno S., Romero-Imbroda J., Vidal-Jordana A., Perez-Miralles F. (2021). Cognitive impairment in multiple sclerosis: Diagnosis and monitoring. Neurol. Sci..

[26] Delgado-Alvarez A., Matias-Guiu J.A., Delgado-Alonso C., Hernandez-Lorenzo L., Cortes-Martinez A., Vidorreta L., Montero-Escribano P., Pytel V., Matias-Guiu J. (2020). Cognitive Processes Underlying Verbal Fluency in Multiple Sclerosis. Front. Neurol..

[27] Blecher T., Miron S., Schneider G.G., Achiron A., Ben-Shachar M. (2019). Association Between White Matter Microstructure and Verbal Fluency in Patients with Multiple Sclerosis. Front. Psychol..

[28] Robinson G., Shallice T., Bozzali M., Cipolotti L. (2012). The differing roles of the frontal cortex in fluency tests. Brain.

[29] Taranu D., Tumani H., Holbrook J., Tumani V., Uttner I., Fissler P. (2022). The TRACK-MS Test Battery: A Very Brief Tool to Track Multiple Sclerosis-Related Cognitive Impairment. Biomedicines.

[30] Ross T.P., Calhoun E., Cox T., Wenner C., Kono W., Pleasant M. (2007). The reliability and validity of qualitative scores for the Controlled Oral Word Association Test. Arch. Clin. Neuropsychol..

[31] Aschenbrenner S., Tucha O., Lange K.W. (2000). Regensburger Wortflüssigkeits-Test (RWT).

[32] Shao Z., Janse E., Visser K., Meyer A.S. (2014). What do verbal fluency tasks measure? Predictors of verbal fluency performance in older adults. Front. Psychol..

[33] Thompson A.J., Banwell B.L., Barkhof F., Carroll W.M., Coetzee T., Comi G., Correale J., Fazekas F., Filippi M., Freedman M.S. (2018). Diagnosis of multiple sclerosis: 2017 revisions of the McDonald criteria. Lancet. Neurol..

[34] Cao H., Peyrodie L., Agnani O., Cavillon F., Hautecoeur P., Donzé C. (2015). Evaluation of an Expanded Disability Status Scale (EDSS) modeling strategy in multiple sclerosis. Med. Biol. Eng. Comput..

[35] Elwood R.W. (1991). The Wechsler Memory Scale-Revised: Psychometric characteristics and clinical application. Neuropsychol. Rev..

[36] Zimmermann P., Fimm B. (1992). Testbatterie Zur Aufmerksamkeitsprufung (TAP).

[37] Petermann F. (2012). Wechsler Adult Intelligence Scale—Fourth Edition (WAIS-IV).

[38] Filser M., Schreiber H., Pottgen J., Ullrich S., Lang M., Penner I.K. (2018). The Brief International Cognitive Assessment in Multiple Sclerosis (BICAMS): Results from the German validation study. J. Neurol..

[39] Snaith R.P. (2003). The Hospital Anxiety And Depression Scale. Health Qual. Life Outcomes.

[40] Penner I.K., Raselli C., Stöcklin M., Opwis K., Kappos L., Calabrese P. (2009). The Fatigue Scale for Motor and Cognition Functions (FSMC): Validation of a new instrument to assess multiple sclerosis-related fatigue. Mult. Scler..

[41] Lix L.M., Keselman J.C., Keselman H.J. (1996). Consequences of Assumption Violations Revisited: A Quantitative Review of Alternatives to the One-Way Analysis of Variance F Test. Rev. Educ. Res..

[42] Conradsson D., Ytterberg C., von Koch L., Johansson S. (2018). Changes in disability in people with multiple sclerosis: A 10-year prospective study. J. Neurol..

[43] Brochet B., Ruet A. (2019). Cognitive Impairment in Multiple Sclerosis with Regards to Disease Duration and Clinical Phenotypes. Front. Neurol..

[44] Portaccio E., Amato M.P. (2022). Cognitive Impairment in Multiple Sclerosis: An Update on Assessment and Management. NeuroSci.

[45] Louapre C., Perlbarg V., Garcia-Lorenzo D., Urbanski M., Benali H., Assouad R., Galanaud D., Freeman L., Bodini B., Papeix C. (2014). Brain networks disconnection in early multiple sclerosis cognitive deficits: An anatomofunctional study. Hum. Brain Mapp..

[46] Rocca M.A., Valsasina P., Hulst H.E., Abdel-Aziz K., Enzinger C., Gallo A., Pareto D., Riccitelli G., Muhlert N., Ciccarelli O. (2014). Functional correlates of cognitive dysfunction in multiple sclerosis: A multicenter fMRI Study. Hum. Brain Mapp..

[47] Akaike S., Okamoto T., Kurosawa R., Onodera N., Lin Y., Sato W., Yamamura T., Takahashi Y. (2023). Exploring the Potential of the Corpus Callosum Area as a Predictive Marker for Impaired Information Processing in Multiple Sclerosis. J. Clin. Med..

[48] Gregorio F.D., Battaglia S. (2024). The intricate brain-body interaction in psychiatric and neurological diseases. Adv. Clin. Exp. Med..

[49] Calabrese M., Agosta F., Rinaldi F., Mattisi I., Grossi P., Favaretto A., Atzori M., Bernardi V., Barachino L., Rinaldi L. (2009). Cortical lesions and atrophy associated with cognitive impairment in relapsing-remitting multiple sclerosis. Arch. Neurol..

[50] Petracca M., Pontillo G., Moccia M., Carotenuto A., Cocozza S., Lanzillo R., Brunetti A., Brescia Morra V. (2021). Neuroimaging Correlates of Cognitive Dysfunction in Adults with Multiple Sclerosis. Brain Sci..

[51] Burggraaff J., Liu Y., Prieto J.C., Simoes J., de Sitter A., Ruggieri S., Brouwer I., Lissenberg-Witte B.I., Rocca M.A., Valsasina P. (2021). Manual and automated tissue segmentation confirm the impact of thalamus atrophy on cognition in multiple sclerosis: A multicenter study. Neuroimage Clin..

[52] Rocca M.A., Amato M.P., De Stefano N., Enzinger C., Geurts J.J., Penner I.K., Rovira A., Sumowski J.F., Valsasina P., Filippi M. (2015). Clinical and imaging assessment of cognitive dysfunction in multiple sclerosis. Lancet Neurol..

[53] Piacentini C., Argento O., Nocentini U. (2023). Cognitive impairment in multiple sclerosis: “classic” knowledge and recent acquisitions. Arq. Neuropsiquiatr..

[54] Elwick H., Topcu G., Allen C.M., Drummond A., Evangelou N., Nair R.D. (2022). Cognitive measures used in adults with multiple sclerosis: A systematic review. Neuropsychol. Rehabil..

[55] Oset M., Stasiolek M., Matysiak M. (2020). Cognitive Dysfunction in the Early Stages of Multiple Sclerosis-How Much and How Important?. Curr. Neurol. Neurosci. Rep..

[56] Elwick H., Smith L., Mhizha-Murira J.R., Topcu G., Leighton P., Drummond A., Evangelou N., Das Nair R. (2021). Cognitive assessment in multiple sclerosis clinical care: A qualitative evaluation of stakeholder perceptions and preferences. Neuropsychol. Rehabil..

[57] Stavrogianni K., Giannopapas V., Kitsos D.K., Christouli N., Smyrni V., Chasiotis A.K., Akrivaki A., Dimitriadou E.M., Tzartos J.S., Tsivgoulis G. (2025). Cognitive Impairment in Newly Diagnosed Patients with Multiple Sclerosis: A Systematic Review of Related Molecular Biomarkers and a Meta-Analysis of Associated Demographic and Disease-Related Characteristics. J. Clin. Med..

[58] Pless S., Woelfle T., Naegelin Y., Lorscheider J., Wiencierz A., Reyes O., Calabrese P., Kappos L. (2023). Assessment of cognitive performance in multiple sclerosis using smartphone-based training games: A feasibility study. J. Neurol..

[59] Ruano L., Branco M., Severo M., Sousa A., Castelo J., Araujo I., Pais J., Cerqueira J., Amato M.P., Lunet N. (2020). Tracking cognitive impairment in multiple sclerosis using the Brain on Track test: A validation study. Neurol. Sci..

[60] Meca-Lallana J.E., Prieto-Gonzalez J.M., Jimenez-Veiga J., Carreon-Guarnizo E., Jimenez-Martin I., Hernandez-Clares R., Sistiaga-Berrondo A., Carles-Dies R., Garcia-Molina E., Cerdan-Sanchez M. (2019). Development and validation of a brief electronic screening test for cognitive impairment in multiple sclerosis (SCI-MS Test). Mult. Scler. Relat. Disord..

[61] National Institute for Health and Care Excellence (NICE) (2022). Multiple Sclerosis in Adults: Management.

[62] Carotenuto A., Costabile T., Pontillo G., Moccia M., Falco F., Petracca M., Petruzzo M., Russo C.V., Di Stasi M., Paolella C. (2022). Cognitive trajectories in multiple sclerosis: A long-term follow-up study. Neurol. Sci..

[63] Chen O.Y., Lipsmeier F., Phan H., Dondelinger F., Creagh A., Gossens C., Lindemann M., de Vos M. (2023). Personalized Longitudinal Assessment of Multiple Sclerosis Using Smartphones. IEEE J. Biomed. Health Inform..

[64] Podda J., Tacchino A., Ponzio M., Di Antonio F., Susini A., Pedulla L., Battaglia M.A., Brichetto G. (2024). Mobile Health App (DIGICOG-MS) for Self-Assessment of Cognitive Impairment in People with Multiple Sclerosis: Instrument Validation and Usability Study. JMIR Form. Res..

[65] Patti F., Amato M.P., Trojano M., Bastianello S., Tola M.R., Goretti B., Caniatti L., Di Monte E., Ferrazza P., Brescia Morra V. (2009). Cognitive impairment and its relation with disease measures in mildly disabled patients with relapsing-remitting multiple sclerosis: Baseline results from the Cognitive Impairment in Multiple Sclerosis (COGIMUS) study. Mult. Scler. J..

[66] DeLuca J., Chiaravalloti N.D., Sandroff B.M. (2020). Treatment and management of cognitive dysfunction in patients with multiple sclerosis. Nat. Rev. Neurol..

[67] Crowe S.F. (1998). Decrease in performance on the verbal fluency test as a function of time: Evaluation in a young healthy sample. J. Clin. Exp. Neuropsychol..

